# Medical residents and eating disorders: an investigation of prevalence and correlates in hospital settings in Damascus

**DOI:** 10.1186/s13030-025-00330-2

**Published:** 2025-07-18

**Authors:** Lujain Nahas, Jameel Soqia, Lama Mohamad, Laila Yakoub Agha, Mehdy Nahas, Bayan Alsaid

**Affiliations:** 1 Faculty of Medicine, Damascus University, Damascus, Syria; 2 Laboratory of Anatomy, Faculty of Medicine, University of Damascus, Damascus, Syria

**Keywords:** Eating disorders, Medical residents, Eating behaviors, BMI, Risk factors

## Abstract

**Objective:**

To study how common are disordered eating behaviors among medical residents in different specialties and how certain preceding factors might contribute to that risk.

**Methods:**

Self-administered questionnaires were administered to residents from each year and specialty using the Eating Attitudes Test-26 (EAT-26) and the Sick, Control, One, Fat, Food (SCOFF) questionnaires. We used binary logistic regression to study the relationship between individuals at high risk and possible triggers of eating disorders.

**Results:**

Among the 399 participants, the risk of disordered eating behavior was 8% using the EAT-26 and 14.3% using SCOFF. There was no difference in the risk according to sex or specialty except for dermatology (*p* = 0.004). BMI was also among the affecting factors (*p* < 0.05), in addition to a positive family and personal history of mental disorders (*p* < 0.001). Recent exposure to stressors, living and marital status did not prove to affect risk.

**Conclusion:**

We found that a percentage of medical residents in different specialties are at greater risk for developing eating disorders and exhibit alarming behaviors related to feeding habits. This risk stems from a number of variables, a few of which were studied in this article. Our results demonstrate a need for better awareness of mental health.

## Background

Eating disorders (EDs) consist of a spectrum of highly variable cases that cause great disruption to quality of life and threatens with high levels of morbidity and psychological distress. However, they can be considered relatively understudied compared to other psychological disturbances [1, 2]. The study of the characteristics of patients with EDs is ongoing, as several neural and chemosensory theories have been tested, showing promise for better understanding and henceforth more effective treatments [3, 4].

The healthcare workforce, including doctors and residents (who are considered doctors in training in hospital settings after graduating medical school), is met with substantial challenges and daily stress. This burden differs in perception among different individuals, but nevertheless, it has been proven that this continuous subjection puts residents at greater risk of psychological distress and susceptibility to psychological disorders [5, 6].

Globally speaking, a 10.4% risk prevalence was estimated in medical students which was identified using EAT-26, this population is generally more prone to stress overload that may precede a disorder risk or exacerbate it [7]. This risk has been studied and was found to have variable correlations with many factors among which are genetics, personality traits and co-morbid mental health conditions, gender and body image and social influence [8]. Syrian literature lacks extensive research in this field, as not much literature is available to generate a reliable recent prevalence number, and despite being included in a recent 2022 meta-analysis that proposed a 22.8% prevalence among adolescents and young adults, this was based on one single study that used results of EAT-26 [9]. To our knowledge, there are no similar studies on the medical community, which creates a gap in the research required to highlight this situation and address it properly in our community. Physicians are at a higher danger to develop mental disorders compared with the general population [10]. Furthermore, residents in hospital settings in Damascus are subjected to a unique set of factors and stressors that may place them at a higher risk for developing these disorders, as they are considered first-line healthcare providers. This observation prompted us to study the risk of eating disorders in a third-world country facing major healthcare setbacks and struggles as one possible outcome in high-risk individuals.

Physicians also put their patients’ needs before their own. This is amplified in emergencies, or life-threatening experiences such as the COVID pandemic and the Syrian war in this population. Furthermore, Physicians are also met with extra-occupational stressors such as family-related or financial burdens [11, 12]. Interestingly, we have also found that there is a knowledge gap for healthcare workers in terms of EDs. While this can result in various forms, one of these results is that ED risk will go unnoticed and they will not be able to identify dangerous eating habits that might result in full disorders [13].

This makes this study a necessity and the first to inspect risk prevalence and further associated factors. We predict a significant level of high-risk individuals among the different specialties and difference in risk prevalence among different groups with higher stress and workload such as surgery and internal medicine.

## Methods and materials

### Study design

This cross-sectional study was conducted in the educational hospitals of the Ministry of Higher Education in Damascus, Syria, which includes the National University, Al Mouasat, Pediatrics, Gynecology and Dermatology hospitals. We aimed to investigate the prevalence of eating disorder risk in a highly susceptible and understudied population to gain better understanding and to raise awareness about these disorders.

### Participants and sample size

Residents who are currently specializing in the aforementioned hospitals and are master students for the ministry of higher education from every specialty and year were asked to fill out a questionnaire administered to them personally from September 2023 to November 2023, informed consent was obtained from participants at the beginning. Participants were selected randomly and were excluded due to missing data and incomplete questionnaires. The necessary sample size was calculated to represent residents’ communities and different specialties, a minimum of 311 participants was found to be representative of the population by a previously published study, further distribution of the sample numbers between specialties and our study’s final sample can be found in Table 1 [14].

### Measurements

The survey included demographic inquiries and two eating disorder risk assessment questionnaires, namely, the Eating Attitude Test-26 (EAT-26) and SCOFF [15, 16]. The aim of using to screening tools was the search for the most accuracy, as both tools are widely used and praised and for the aid of future research on the comparison of efficacy of screening to determine more specific tools. Before starting the questionnaires, participants were asked to answer demographic questions concerning recent familial or social stressors, utilization of mental health services and medications, current residency year and specialty, living circumstances, and medical and family histories of mental and eating disorders. Recent familial or social stressors, utilization of mental health services and medications and medical and family histories of mental and eating disorders were predefined with yes/no responses while current marital status was predefined with single/ married and housing was a choice between family and dorms. Body mass index (BMI) was calculated by using current height and weight provided by each participant.

Both questionnaires used in our study helped identify a primary risk of EDs:

The Eating Attitudes Test-26 (EAT-26): a self-administered questionnaire that is used for the primary detection of individuals with high ED risk. This questionnaire mainly screens for the risk of binge eating disorder (BED), anorexia nervosa (AN) and bulimia nervosa (BN). It comprises of three sections, the first section includes demographic information such as age, gender and current height and weight. The second section consisted of 26 questions. Each item is scored on a six-point scale ranging from always to never. The questions can be grouped into three main topics: dieting questions, bulimia and food preoccupation and oral control. The questionnaire’s results do not allow for diagnosis of EDs but the identification if its risk as follows: the total sum of the second section ranges from 0 to 78. The cutoff point is a score of 20 or higher and is classified as high risk [15]. The third part asked about behavioral weight-control patterns, including binge eating episodes, self-induced vomiting, laxative diet pill or diuretic use and excessive exercise to control shape and weight, along with a weight loss of more than 9 kg over the past 6 months. Each behavior was scored as a frequency (never, once a month or less, 2–3 times a month, once a week, 2–6 times a week and once or more in a day) while weight loss was predefined as a yes/no. This section assessed each behavior separately and set a frequency at which the behavior should be clinically investigated (binge eating if 2–3 times a month or more, forced vomiting and use of diuretics and laxatives at any rate, over 60 min of exercise if only practiced once a day or more). The validated Arabic version of the EAT-26 questionnaire was used in this study [17]. Cronbach alpha for our sample was calculated at 0.85 which demonstrates strong internal integrity.

The SCOFF questionnaire is another widely used self-administered questionnaire for ED risk screening [16]. Five questions are answered “yes” or “no”, and a score of 2 or more is considered high risk [18]. A validated Arabic version was also used in our study [19].

S – Do you make yourself Sick because you feel uncomfortably full?

C – Do you worry you have lost Control over how much you eat?

O – Have you recently lost more than One stone (6.35 kg) in a three-month period?

F – Do you believe yourself to be Fat when others say you are too thin?

F – Would you say Food dominates your life?

### Ethical considerations

Participants were asked to complete a printed self-administered questionnaire. Informed consent was obtained, and the participants were informed that their data would not be shared outside this study. The study was approved by the ethical committee of Damascus University (ID number: 241023-130, session 8 on 24/10/2023) and was performed in accordance with the ethical standards of the Helsinki II Declaration about informed consent, voluntariness and anonymity. The data collection tools contained no identifying information and therefore kept the individual responses confidential. A disclaimer indicated that participation in the study was purely voluntary and that they could choose to exit whenever they wanted.

### Data analysis

All analyses were performed using IBM SPSS Statistics for Windows, version 25 (IBM Corp., Armonk, N.Y., USA). Self-administered questionnaires were used to collect the data. Prevalence rates were calculated based on the recommended cut-offs for Eat-26 and SCOFF. Separate logistic regressions were used to determine predictors of high ED risk according to the Eat-26 and SCOFF scores. In each regression, sex, BMI, specialty, recent stressors, place of residence, age, year of specialty, marital status, visiting a health care professional, taking psychiatric medication, personal positive mental history and positive family history of eating disorders were entered as predictors in step 1.

**Table 1:** Participants’ specialization distributions and sample calculations

Specialization	Total number of residents	Minimum required number	Sample size for our study
Pediatrics	120	24	56
Hematology	20	4	5
Obstetrics and Gynecology	42	7	20
Pediatric Surgery	7	2	11
Thoracic Surgery	24	5	5
Plastic Surgery	26	5	5
Orthopedics	49	10	13
Radiology	243	46	47
Clinical Pathology	56	10	10
Pulmonology	29	6	6
Gastroenterology	29	6	14
Anesthesiology	87	15	15
Dermatology	58	11	19
Cardiac Surgery	42	9	9
Nephrology	25	5	7
General Internal Medicine	235	45	45
Cardiology	26	5	5
Neurology	25	5	6
Oncology	36	7	7
Rheumatology	23	5	5
Endocrinology	18	4	5
Laboratory	53	11	11
Emergency Medicine	10	2	2
Psychiatry	45	9	9
Infectious Diseases Medicine	19	4	4
General Surgery Medicine	68	13	13
Urinary Surgery	33	7	7
Neurosurgery	25	5	10
Vascular Surgery	29	6	6
Ophthalmology	41	8	9
Otolaryngology	46	9	12
Radiotherapy	5	1	1
**Total**	**1595**	**311**	**399**

## Results

### Demographic characteristics

Data from 399 residents were collected. 50.1% were males. The age of the participants ranged from 24 to 37 years, with a mean age of 25.86 years (SD = 1.51). The BMI of the participants ranged from 14.38 to 56.69, with a mean BMI of 23.44 (SD = 3.22). The majority of the participants were single (81.7%) and reported being subjected to recent stressors (72.7%). Additionally, 52.1% of the residents lived with their family rather than with university dorms (47.9%). Only 11 (2.8%) were currently seeking help from a healthcare specialist, and 22 (5.5%) were taking psychiatric medication. Furthermore, 29 (7.3%) reported a positive mental health history (i.e. a previous diagnosis of a psychological disorder) and 36 (9%) reported a positive family history of eating disorders. Further details, characteristics, and differences among the participants are outlined in Table 2.

### Risk of eating disorders

Overall, the mean score of the EAT-26 was 7.46 (SD = 7.49); after applying a cutoff point of 20, thirty-two (8%) residents had a high risk of eating disorders. Moreover, the mean SCOFF score was 0.55 (SD = 0.86); after applying a cutoff point of 2, fifty-seven (14.3%) residents had a high risk of eating disorders. 32 specialties were grouped into 13 groups (internal medicine, surgery, radiology, otolaryngology, ophthalmology, pathology anesthesiology, emergency medicine, gynecology and obstetrics, laboratory, radiotherapy, dermatology and pediatrics). The highest risk using the EAT-26 was found in Internal Medicine (2.7%) and in Surgery when using SCOFF (3.5%). The second group with the highest risk prevalence included Pediatrics, Dermatology and Radiology (1.7%). Of the 290 people who reported being subjected to recent stressors, 10 were only seeing a physician (3.4%). Interestingly, the same was true for residents who had a high risk of EDs: one patient who achieved a high score on SCOFF was seeing a physician (1.75%), while one patient from the EAT-26 high score group (3.1%) was seeing a physician.

Regarding the behavioral part of the EAT-26 questionnaire, we only highlighted frequency of every behavior that required clinical attention, which was as follows: 26.2% reported binge eating behaviors at a frequency between 2 and 3 times a month and once a day or more. Induced vomiting was reported between once a month or less and once a day or more at a percentage of 3.4%. The percentage of residents who used laxatives and/or diuretics was 5.7%, while excessive exercise was considered alarming only if it was practiced once a day or more at 1.5%. Additionally, 14.7% of the participants were positive for a recent weight loss of 9 kg in the past 6 months. Further elaboration of these results can be found in Table 3.

### Predictors and high risk of eating disorders

Table 4 shows the odds ratio between males and females with a high risk of eating disorders. The table indicates that 8% of males and 8% of females have a high risk of eating disorders. The odds ratio between males and females was 1.00 (95% CI: 0.48–2.07), as there were no significant differences between males and females. With respect to specialty, no significant relationship was observed with any of the studied specialties except for Dermatology, where a value of *p* = 0.004 according to binary logistic regression suggested the only present importance.

A high risk of eating disorders was associated with a greater BMI than a low risk of eating disorders (24.77 > 23.32) (t(397) =-2.004, *P* = 0.046), with a 95% CI of [-2.86, -0.02].

By further investigating this relationship, we obtained ORs of 1.08 (95% CI: 1.00-1.16) for EAT risk and 1.09 (95% CI: 1.02–1.02) for SCOFF.

Table 5 presents the results of binary regression analysis aimed at determining the adjusted odds ratios for a high risk of eating disorders associated with various factors. The adjusted odds ratios, along with their corresponding 95% confidence intervals, provide insights into the potential associations between these variables and the likelihood of individuals being at a high risk of eating disorders. Notably, positive family and personal histories of eating disorders were significantly associated with increased risk, as indicated by odds ratios of 4.09 (95% CI: 1.57–10.61) for EAT risk, 3.53 (95% CI: 1.65–7.55) for family history and 3.45 (95% CI: 1.29–9.22) for personal history. Other variables, while showing trends, did not reach statistical significance based on the confidence intervals.

**Table 2:** Residents’ sample characteristics (all participants are Syrian)

Variables		Frequency (*n*)	Percent (%)	Means (SDs)
Gender	Males Females	200 199	50.1% 49.9%	-
Age	24–37 years	-	-	25.86 (1.51)
BMI	14.38–56.69			23.44 (3.92)
BMI classifications	Underweight	23	5.7%	
	Normal	250	62.6%	
	Overweight	102	25.5%	
	Obese (class 1, 2 and 3)	24	6.0%	
Marital Status	Single	326	81.7%	-
Recent Stressors	Yes	290	72.7%	-
Where do you live	With family	208	52.1%	-
Year of Residency	First	219	54.9%	
	Second	89	22.3%	
	Third	49	12.3%	
	Fourth	32	8.0%	
	Fifth	7	1.8%	
	Sixth	3	0.8%	
Currently Seeing a Healthcare Specialist	yes	11	2.8%	-
Currently Taking any Psychiatric Medication	Yes	22	5.5%	-
Personal Mental Health History	Yes	29	7.3%	-
Family History of Eating Disorders	Yes	36	9.0%	

**Table 3:** The third section of the EAT-26 demonstrating the frequency of the behaviors that require clinical attention (the latter are marked with*)

Variables for EAT-26 *N* = 399		*n* (%)
Binge Eating Episodes	Never ≤ Once per month 2–3 times a month* Once a week* 2–6 times a week* ≥ Once a day*	197 (49.3) 95 (23.8) 41(10.2) 43 (10.7) 11 (2.7) 12 (3.0)
Vomiting for weight shape or control	Never ≤ Once per month* 2–3 times a month* Once a week* 2–6 times a week* ≥ Once a day*	385 (96.4) 3 (0.7) 1 (0.2) 1 (0.2) 9 (2.2) 0
Use of laxatives, diet pills or diuretics for weight control or shape	Never ≤ Once per month* 2–3 times a month* Once a week* 2–6 times a week* ≥ Once a day*	376 (94.2) 4 (1.0) 4 (1.0) 1 (0.2) 4 (1.0) 10 (2.5)
Exercise ≥ 60 min/day for weight loss or control	Never ≤ Once per month 2–3 times a month Once a week 2–6 times a week ≥ Once a day*	278 (69.6) 50 (12.5) 18 (4.5) 26 (6.5) 21`(5.2) 6 (1.5)
Weight loss ≥ 20 lbs. (9 kg)	Yes	59 (14.7)

**Table 4:** The odds ratio between males and females with high risk of eating disorders (calculated using both EAT-26 and SCOFF risk at a time)

	Males (Referenced)	Females	OR (95% CI)
High risk of eating disorders	16 (8% of total males)	16 (8% of total females)	1.00 (0.48–2.07)

* *p* < 0.05, ** *p* < 0.001

Unadjusted odds ratios were used in this table

**Table 5:** Shows the results of binary logistic regression to determine the adjusted odds ratio for a high risk of eating disorders according to multiple variables (* *p* < 0.05, ** *p* < 0.001)

	high risk of eating disorders (using EAT-26 risk)	high risk of eating disorders (using SCOFF risk)	Estimated effect size (using Nagelkerke *R* square)
Gender (males is referenced)	1.0 (0.48–2.07)	1.58 (0.89–2.8)	<0.01
Age	0.97 (0.75–1.27)	1.03 (0.86–1.23)	<0.01
BMI	1.01 (1.02–1.17)*	1.09 (1.02–1.17)*	0.15
Recent Stressors (not having is referenced)	0.95 (0.42–2.13)	1.91 (0.93–3.94)	< 0.01
Where do you live (family house is referenced)	1.12 (0.52–2.42)	1.01 (0.57–1.77)	< 0.01
Marital Status (married is referenced)	0.89 (0.33–2.42)	1.06 (0.5–2.21)	< 0.01
Visiting a Health care professional (not is referenced)	0.31 (0.02–3.98)	0.59 (0.07–4.27)	< 0.01
Taking Psychiatric Medication (not taking is referenced)	2.08 (0.46–9.27)	2.39 (0.89–6.41)	0.12
Personal positive mental history. (no history is referenced)	3.45 (1.29–9.22)**	0.95 (0.32–2.86)	0.17
Positive family history of eating disorders. (no history is referenced)	4.09 (1.57–10.61)**	3.53 (1.65–7.55)**	0.25

## Discussion

This cross-sectional study focused on the paucity of research concerning the status and prevalence of the ED spectrum risk in the Syrian medical community. We met with residents who were part of the higher education ministry in Damascus and asked them to fill self-administered questionnaires.

Initially, a percentage of high-risk populations reached 8% and 14.3% according to the EAT-26 and the SCOFF questionnaires respectively. In general, the result is consistent with the global percentage of the risk estimated in medical students of 10.4% [7] but is considerably lower than the results from a meta-analyses of a number of other Middle-Eastern counties which studied risk prevalence in adolescence (total percentage of 22.9%) [9]. We propose that this may be due to the high level of resilience and adaptation that these residence have developed during the urgent times they have faced in their career, as physicians were shown to show more resilience than the general population. Furthermore, physicians in Damascus had to stay on top of their responsibilities during the COVID pandemic and the Syrian war [20].

This population has also not been widely exposed to western ideal beauty standards through media at an earlier more impressionable age as middle-eastern counties have only recently been vastly influenced by new coming beauty standards. The risk percentage varied between specialty groups, the groups with the highest risk were surgery and internal medicine. This difference is due to the various conditions that residents face in different specialties and how they perceive the pressure and conditions they are met with [5]. In contrast with the aforementioned research and prevailing consensus that females are more prone to developing EDs [21], our study did not yield evidence of a significant correlation between sex and susceptibility to developing eating disorders. Several studies we found have shown that our studied risk may not always be moderated by sex [22], and a study in India reported similar results between females and males [23]. Thus, our findings, while raising questions, are credible. Interestingly, it is noted that females with ED symptoms tend to under-report those symptoms which highlighted the need of tools that are especially directed for these females [24]. This could also be used to explain the difference. Despite this, we propose an alternative interpretation based on the exclusive association between susceptibility and the field of dermatology. Traditionally categorized as a specialty with minimal stress and predominantly populated by women (our sample included 2 males, neither of whom exhibited high susceptibility to EDs. This can be used to solidify the general argument of female abundance. However, despite the unequal distribution of susceptibility, eating disorders in males have gained noticeable attention, as 1 in 4 cases of anorexia nervosa (AN) and Bulimia nervosa (BN) are observed in males [25].

In terms of subjection to stress, residents in government hospitals face a substantial burden of stress in their daily duties, and this stress varies widely among individuals in perception and seems to affect them based on subjective perception as some sources of stress include academic workload and continuous exposure to human suffering and so employment in hospital setting and being constantly exposed to various burdens inevitably places healthcare workers at higher risk of developing many psychological disorders [5]. However, despite these differences, no association was detected between exposure to recent stressors and the risk of eating disorders, and there was no further correlation between different medical specialties and the risk of eating disorders, despite their variations. This in turn could also be attributed to the development of a certain level of adaptability in addition to the stage of medical knowledge residents have built up and their access to healthcare departments. This, in turn, brings us to the subsequent factor that has proven to be significant: family history. Interestingly, this factor is interchangeable: while children born to a parent with an ED are almost 3–5 times more susceptible to risk of their own development [26], parents of children with EDs may start having personal concerns regarding weight and body image, thus triggering a new risk [27]. However, regardless of the presumably hereditary nature of EDs, this process is not customable to a certain ED and may be associated with an increased risk of other psychological disturbances, such as mood and anxiety disorders and autistic spectrum disorders [28, 29].

Moreover, despite the evident correlation between a personal positive history of mental health and the risk of EDs, our study suggested otherwise. However, as we found many studies observing and identifying multiple comorbid conditions that may cause new EDs to move into motion, we urge that more elaborate studies that may prove that this relationship is significant [8].

The final variable with robust significance was BMI, which can be traced back to childhood weight, as both lower and higher BMIs are associated with different ED risks [8], as there is strong evidence stating that the probability of a young adult with BMI values higher than normal is 2.45 times more prone to develop a distorted relationship with food [30]. Even if more individuals with overweight and obesity display a greater tendency to exhibit disordered eating, underweight individuals cannot be marginalized, as almost 6% of ED patients are underweight [31]. In our sample, residents may use eating as a way to blow off steam and this might turn into pathological eating habits. On the contrary, alarming food habits can also be formed in a restrictive manner as some specialties like surgery and pediatrics are on call most of their time, which might hinder them from getting used to proper eating habits therefore triggering a possible risk.

### Limitations

Given the cross-sectional nature of the study and the use of self- administered questionnaires that screened for risk only, more longitudinal investigations and research are required to further emphasize the role of eating risk predictors. Furthermore, we advise that future studies that use screening tools for risk are followed by specialized interviews to achieve diagnosis of probable disorders and to start with treatment as soon as possible. Our study was also conducted during wartime conditions, which may bring bias to the results. Finally, we also urge that future research takes place in several private and governmental hospitals and healthcare centers so that it encompasses more diversity and different residents subjected to different conditions.

## Conclusions

This study aimed to identify the primary prevalence of a high risk of disordered eating behavior, with initial results of 8% using the EAT-26 and 14.3% using SCOFF. Moreover, the identification of the most relevant risk factors for developing EDs is just as important as diagnosis and requires more research. Our results demonstrated that BMI and a positive family and personal history of eating disorders were among the most important identifiable factors. These correlations emphasize the integration and complexity of triggering factors, and our results urge deeper understanding and research and further support for healthcare workers, especially residents.

## Data Availability

The data that support the findings of this study are available upon request from the corresponding author (L.N.). Permission to use Table 1 was acquired from the corresponding author (J.S.).
